# Artificial neural network approach to predict surgical site infection after free-flap reconstruction in patients receiving surgery for head and neck cancer

**DOI:** 10.18632/oncotarget.24468

**Published:** 2018-02-09

**Authors:** Pao-Jen Kuo, Shao-Chun Wu, Peng-Chen Chien, Shu-Shya Chang, Cheng-Shyuan Rau, Hsueh-Ling Tai, Shu-Hui Peng, Yi-Chun Lin, Yi-Chun Chen, Hsiao-Yun Hsieh, Ching-Hua Hsieh

**Affiliations:** ^1^ Department of Plastic and Reconstructive Surgery, Kaohsiung Chang Gung Memorial Hospital and Chang Gung University College of Medicine, Kaohsiung, Taiwan; ^2^ Department of Anesthesiology, Kaohsiung Chang Gung Memorial Hospital and Chang Gung University College of Medicine, Kaohsiung, Taiwan; ^3^ Department of Neurosurgery, Kaohsiung Chang Gung Memorial Hospital and Chang Gung University College of Medicine, Kaohsiung, Taiwan; ^4^ Center for Vascularized Composite Allotransplantation, Chang Gung Memorial Hospital, Taoyuan, Taiwan

**Keywords:** artificial neural network (ANN), free-flap reconstruction, head and neck cancer, logistic regression (LR), surgical site infection (SSI)

## Abstract

**Background:**

The aim of this study was to develop an effective surgical site infection (SSI) prediction model in patients receiving free-flap reconstruction after surgery for head and neck cancer using artificial neural network (ANN), and to compare its predictive power with that of conventional logistic regression (LR).

**Materials and methods:**

There were 1,836 patients with 1,854 free-flap reconstructions and 438 postoperative SSIs in the dataset for analysis. They were randomly assigned tin ratio of 7:3 into a training set and a test set. Based on comprehensive characteristics of patients and diseases in the absence or presence of operative data, prediction of SSI was performed at two time points (pre-operatively and post-operatively) with a feed-forward ANN and the LR models. In addition to the calculated accuracy, sensitivity, and specificity, the predictive performance of ANN and LR were assessed based on area under the curve (AUC) measures of receiver operator characteristic curves and Brier score.

**Results:**

ANN had a significantly higher AUC (0.892) of post-operative prediction and AUC (0.808) of pre-operative prediction than LR (both *P*<0.0001). In addition, there was significant higher AUC of post-operative prediction than pre-operative prediction by ANN (p<0.0001). With the highest AUC and the lowest Brier score (0.090), the post-operative prediction by ANN had the highest overall predictive performance.

**Conclusion:**

The post-operative prediction by ANN had the highest overall performance in predicting SSI after free-flap reconstruction in patients receiving surgery for head and neck cancer.

## BACKGROUND

Although success rates of microvascular free-flap reconstruction in patients undergoing head and neck cancer surgery are very high [1, 2], the rate of surgical site infections (SSIs) still ranges from 10 to 40% [3–5]. This is probably because of the complex anatomic structure of the head and neck as well as the contaminated environment near the regions of mouth and throat. Aggressive surgical procedures and cancer ablation destruct the barrier of oral mucosa and expose the wound directly to bacteria from the mouth and pharynx. A free-flap reconstruction further complicates the situation with the extended operation time and additional risk of exposure to contaminants [6]. Furthermore, salivary leakage and wound bed contamination aggravates the occurrence of SSIs [7].

Many predictive factors associated with SSIs in patients with head and neck cancer have been proposed. They include higher tumor stage [8, 9], pre-operative chemotherapy [4], radiotherapy [10–12], presence of comorbidity [13–16], American Society of Anesthesiologists (ASA) score [17, 18], concurrent neck dissection [9, 11, 19], low hemoglobin (Hb) level [19], low serum albumin concentration [14, 20, 21], perioperative blood transfusion [22], type of the flap [3, 17, 23] and operation time [17]. In multivariate logistic regression (LR) analysis of 376 elderly oral cancer patients, Ma et al. identified six parameters independently associated with the occurrence of SSI. They were: body mass index, diabetes mellitus (DM), ASA score, Adult Comorbidity Evaluation-27 score, operation time, and reconstruction with pectoralis major myocutaneous flaps or free flaps [17]. In a multivariate analysis of 197 patients who underwent head and neck reconstructive surgery, Kamizono et al. reported hypoalbuminemia, reconstruction with vascularized bone transfer, and a poor ASA score were significant risk factors for SSIs [20]. In a review of 1,693 chart records of oral cavity cancer patients, Liu et al. described that DM, perioperative blood transfusion, reconstruction with free flap or pectoris major myocutaneous flap, and post-operative serum albumin level were independent factors associated with SSIs [21]. In a study of 276 cases of free-flap reconstruction for head and neck surgery, Karakida et al. concluded that risk factors for SSIs were a long operation time and poor host immune performance status [3]. Notably, these predictive factors varied and even contradicted each other among studies. For example, multivariate analysis by Lee et al. has shown that a history of radiation carried a 2.85-fold of risk for SSIs after head and neck surgery [10]; however, such risk factor could not be identified in multivariate analysis by Kamizono et al. [20]. In addition, age has been deemed as a definite risk factor for post-operative SSIs. However, in patients with head and neck cancer, age *per se* has not been confirmed as a factor for SSIs in many studies [11, 17, 24]. Furthermore, for patients undergoing head and neck reconstruction, long operation time [20], blood loss [20], tumor location and tumor size [17], and pre-operative radiotherapy [17] were not identified as risk factors. This evidence may indicate a complex relationship of the occurrence of SSI and various patient situations with many risk factors in the patients receiving free-flap reconstruction after head and neck cancer surgery.

Post-operative SSI can lead to vessel thrombosis and eventual flap loss. However, there is a lack of research on risk prediction of SSI in individual patients receiving free-flap reconstruction after head and neck cancer surgery. Most previous prediction models were developed using univariate or multivariate LR analysis [25]. With interpretability of model parameters and ease of use, LR can generate excellent models and serve as a commonly accepted statistical tool. However, LR analysis is based on assumptions of linear relationship between response and explanatory variables, normal distribution of response variables, and homogeneity of variances of the error terms. If some of the above assumptions are not satisfied in actual data, the model cannot be used as it could have considerable errors [26]. In addition, the artificial neutral network (ANN) is constructed from a set of neurons, such as those found in a brain network, that exchange signals with each other via an interconnected network. Each connection has a numeric weight that can be adjusted during training of the network, making the system adaptive to input patterns and capable of revealing previously unknown relationships between given input and output variables [27, 28]. ANN is one of most suitable method to sort out a complex problem without any assumptions. ANN finds the form of relationship, which is not necessarily linear, and has no limitation on the form of relationship between response and predictor variables. Furthermore, it has a high probability of finding the correct solution in ANN, even if a part of network layers is deleted or works incorrectly [26, 29]. Under the hypothesis that ANN may provide a better predictive power of SSI than LR in patients receiving free-flap reconstruction after head and neck cancer surgery. The aim of this study is to develop an effective SSI prediction model using ANN based on comprehensive patient epidemiologic data, disease characteristic, and operative data and compare its predictive power with that of LR.

## RESULTS

### Subject and data preparation

There were 1,854 reconstructions enrolled in the dataset for analysis, which included 1,298 reconstructions in the training set and 538 reconstructions in the test set (Table 1). Of these 1,854 reconstructions in the dataset, there were SSIs in 438 reconstructions (23.6%): 310 (23.6%) and 128 (23.8%) SSIs in the training set and test set, respectively. In the data preparation, because the distribution pattern between Hb and Hct is very similar, only one of these two variables (i.e. Hb) was selected for further classification to prevent inclusion of duplicate parameters. The category of sex was not included as a variable for further classification because there was a very small population of women.

**Table 1:** Categorical variables of patient epidemiologic data, disease characteristic, and operative data

Variables	Status	Total (n=1,854)	Wound Infection		
		n (%)	Yes (n=438)	No (n=1,416)	*P*-value
**Sex**	Male	1,770 (95.5%)	418	1,352	1.00
	Female	84 (4.5%)	20	64	
**Tumor stage**	A. (T0, N=0, M=0)	176 (9.5%)	45	131	<0.01
	B. (T1, N=0, M=0)	208 (11.2%)	30	178	
	C. (T2, N=0, M=0)	313 (16.9%)	46	267	
	D. (T3, N=0, M=0)	87 (4.7%)	27	60	
	E. (T4, N=0, M=0)	450 (24.3%)	102	348	
	F. (N>0, M=0)	610 (32.9%)	181	429	
	G. (M=1)	10 (0.5%)	7	3	
**Tumor location**	Simple reconstruction	161 (8.7%)	44	117	<0.01
	Lip, gum, buccal, palate	961 (51.8%)	190	771	
	Mouth floor, tongue, trigon, tongue base, tonsil	504 (27.2%)	121	383	
	Oro- or hypo-pharyngeal	228 (12.3%)	83	145	
**Primary/Recurrent**	Simple reconstruction	161 (8.7%)	45	116	0.15
	Primary	1,104 (59.3%)	268	836	
	Recurrent	589 (31.8%)	125	464	
**Betalnut hx**	Yes	1,620 (87.4%)	388	1232	0.41
	No	234 (12.6%)	50	184	
**Smoking hx**	Yes	1,637 (88.3%)	393	1244	0.31
	No	217 (11.7%)	45	172	
**Alcohol hx**	Yes	1,540 (88.3%)	380	1160	0.02
	No	314 (16.9%)	58	256	
**DM**	Yes	340 (18.3%)	99	241	0.01
	No	1,514 (81.7%)	339	1,175	
**HTN**	Yes	513 (27.7%)	126	387	0.58
	No	1,341 (72.3%)	312	1029	
**CVA**	Yes	32 (1.7%)	5	27	0.40
	No	1822 (98.3%)	433	1,389	
**Heart disease**	Yes	100 (5.4%)	15	85	0.04
	No	1,754 (94.6%)	423	1,331	
**Liver disease**	Yes	97 (5.2%)	26	71	0.46
	No	1,757 (94.8%)	412	1,345	
**Kidney disease**	Yes	26 (1.4%)	9	17	0.24
	No	1,828 (98.6%)	429	1,399	
**Radiotherapy**	Yes	597 (32.2%)	155	442	0.11
	No	1,257 (67.8%)	283	974	
**Chemotherapy**	Yes	570 (30.7%)	156	414	0.01
	No	1,284 (69.3%)	282	1,002	
**Vein grafting**	Yes	25 (1.3%)	6	19	1.00
	No	1,829 (98.7%)	432	1,397	
**Opposite recipient vessel**	Yes	46 (2.5%)	16	30	0.08
	No	1,808 (97.5%)	422	1,386	
**Anastomosed vessels**	1A1V	1,456 (78.5%)	351	1,105	0.43
	1A2V	388 (20.9%)	86	302	
	2A2V	10 (0.5%)	1	9	
**OP doctors**	OP doctor 1	254 (13.7%)	50	204	0.04
	OP doctor 2	291 (15.7%)	66	225	
	OP doctor 3	206 (11.1%)	52	154	
	OP doctor 4	117 (6.3%)	27	90	
	OP doctor 5	67 (3.6%)	23	44	
	OP doctor 6	475 (25.6%)	132	343	
	OP doctor 7	60 (3.3%)	10	50	
	OP doctor 8	289 (15.6%)	62	227	
	Other doctors	95 (5.1%)	16	79	
**Re-open**	Yes	101 (5.4%)	47	54	<0.01
	No	1,753 (94.6%)	391	1,362	
**Flap**	Anterolateral thigh flap	1,550 (83.6%)	365	1,185	0.53
	Free fibula flap	149 (8.0%)	42	107	
	Free forearm flap	50 (2.7%)	11	39	
	Anteromedial thigh flap	60 (3.2%)	11	49	
	Free style perforator flap	36 (1.9%)	6	30	
	Medial sural artery perforator flap	9 (0.5%)	3	6	

### Univariate logistic regression analysis

To estimate the potential risk factors of SSI, univariate LR analysis was performed in the entire parameter samples. The result indicated eight potential risk factors of categorical variables, including tumor stage, tumor location, alcohol drinking history, DM, heart disease, pre-operative chemotherapy, operative surgeon, and re-open (Table 1), as well as 14 potential risk factors of continuous variables, including BMI, RBC, WBC, Hb, Hct, percentage of neutrophil, albumin, glucose, Cr, Na, flap length, operative time, and the units of transfused packed RBC or plasma (Table 2).

**Table 2:** Continuous variables of patient epidemiologic data, disease characteristic, and operative data

Variables	Total (n=1,854)	Wound Infection		
	Median (IQR)	Yes (n=438)	No (n=1,416)	*P*-value
**Age (years)**	54 (14.0)	54 (14)	55 (13)	0.31
**BMI (kg/m**^2^**)**	23.6 (5.4)	23.3 (5.6)	23.7 (5.4)	0.03
**RBC (10**^6^**/uL)**	4.6 (0.9)	4.5 (1)	4.6 (0.8)	<0.01
**WBC (10**^3^**/uL)**	7 (3.3)	7.6 (3.8)	6.9 (3)	<0.01
**Hb (g/dL)**	13.9 (2.6)	13.5 (3.2)	14 (2.3)	<0.01
**Hct (%)**	41.4 (7.1)	40.7 (8.7)	41.6 (6.7)	<0.01
**Neutrophil (%)**	67.2 (12.6)	69.2 (13.0)	67.1 (12.6)	<0.01
**Platelets (10**^3^**/uL)**	230 (94)	236.5 (105.3)	228 (90.8)	0.06
**INR**	1 (0.1)	1 (0.1)	1 (0.1)	0.19
**Albumin (g/dL)**	4.20 (0.3)	4.0 (0.3)	4.2 (0.2)	<0.01
**Glucose (mg/dL)**	113 (32)	116.4 (34.5)	112 (27.1)	0.03
**K (mEq/L)**	4.1 (0.5)	4.1 (0.6)	4.1 (0.5)	0.29
**BUN (mg/dL)**	13 (6)	13.8 (6)	13 (6)	0.57
**Cr (mg/dL)**	0.9 (0.3)	0.9 (0.3)	0.9 (0.3)	<0.01
**Na (mEq/L)**	139 (4)	139 (4)	140 (3)	<0.01
**AST (U/L)**	24 (11)	25 (10)	24 (10)	0.05
**ALT (U/L)**	23 (16)	24 (17)	23 (16)	0.10
**Flap length (cm)**	20 (10)	20 (10)	18 (9)	<0.01
**OP time (hours)**	7.2 (2.8)	7.5 (2.7)	7.1 (2.8)	0.01
**OP experience (years)**	5.7 (9.9)	5.7 (8.8)	5.7 (10.8)	0.36
**Transfusion – whole blood (U)**	0 (0)	0 (0)	0 (0)	0.23
**Transfusion – packed RBC (U)**	0 (2)	0 (2)	0 (0)	<0.01
**Transfusion - plasma (U)**	0 (0)	0 (0)	0 (0)	<0.01
**Transfusion - platelets (U)**	0 (0)	0 (0)	0 (0)	0.26

### Multivariate logistic regression model

In the multivariate LR model, the significant variables developed from the univariate analysis were used for stepwise elimination of the non-significant variables to obtain final multivariate regression models, which included 14 and 20 independent risk factors of SSI at pre-operative and post-operative stages, respectively (Table 3). At the pre-operative stage, SSI was associated with DM, pre-operative radiotherapy, tumor location, tumor stage, WBC, neutrophil percentage, Cr, heart disease, primary tumor, albumin, CVA, and recurrent tumor. At the post-operative stage, additional six variables (re-open, op doctor 5, amount of transfused packed RBC, operative time, flap length, and glucose) were associated with the occurrence of SSI, but pre-operative radiotherapy was not identified as an independent risk factor for SSI at the post-operative prediction. As shown in Table 4, the accuracy, sensitivity, and specificity of LR in the pre-operative prediction of the training set were 72.64%, 15.69%, and 95.43%, respectively. The accuracy, sensitivity, and specificity of LR in post-operative prediction of the training set were 72.49%, 20.48%, and 93.30%, respectively. In the test set, the accuracy, sensitivity, and specificity of LR in the pre-operative and post-operative prediction were 72.3 ± 0.7%, 14.4 ± 0.8%, and 95.4 ± 0.2% as well as 72.7 ± 0.5%, 22.1 ± 0.8%, and 93.3 ± 0.3%, respectively.

**Table 3:** The independent risk factors identified at pre-operative and post-operative prediction from the multivariate logistic regression model

Pre-op prediction		Post-op prediction	
Independent Variables	Coefficient	Independent Variables	Coefficient
(Intercept)	3.6323	(Intercept)	1.9920
DM	0.5359	Re open	1.0174
Radiotherapy	0.3689	OP doctor 5	0.4894
Tumor location	0.2642	DM	0.4011
Tumor stage	0.1958	Radiotherapy	0.3838
WBC	0.0785	Tumor location	0.2680
Neutrophil	-0.0109	Tumor stage	0.1831
Cr	-0.2562	RBC	0.1578
Heart disease	-0.7186	Transfusion – packed RBC (U)	0.0915
Primary tumor	-0.9867	WBC	0.0761
Albumin	-1.0778	OP time	0.0601
CVA	-1.1050	Flap length (cm)	0.0158
Recurrent tumor	-1.1751	Glucose	0.0020
		Neutrophil	-0.0141
		Cr	-0.2108
		Heart disease	-0.6901
		Primary tumor	-0.9955
		CVA	-1.0018
		Albumin	-1.0731

**Table 4:** The accuracy, sensitivity, and specificity of LR and ANN from the pre-operative and post-operative prediction for the training set and test set

			Pre-op prediction		Post-op prediction	
			Train	Test	Train	Test
**Surgical site infection**	**LR**	**Accuracy**	72.64%	72.3±0.7%	72.49%	72.7±0.5%
		**Sensitivity**	15.69%	14.4±0.8%	20.48%	22.1±0.8%
		**Specificity**	95.43%	95.4±0.2%	93.30%	93.3±0.3%
	**ANN**	**Accuracy**	81.00%	77.8±0.4%	88.37%	75.7±0.6%
		**Sensitivity**	60.90%	61.4±0.8%	71.28%	67.0±1.5%
		**Specificity**	89.04%	89.0±0.4%	95.21%	95.2±0.2%

LR=logistic regression; ANN=artificial neural networks.

### Artificial neural network model

**Figure 1:**
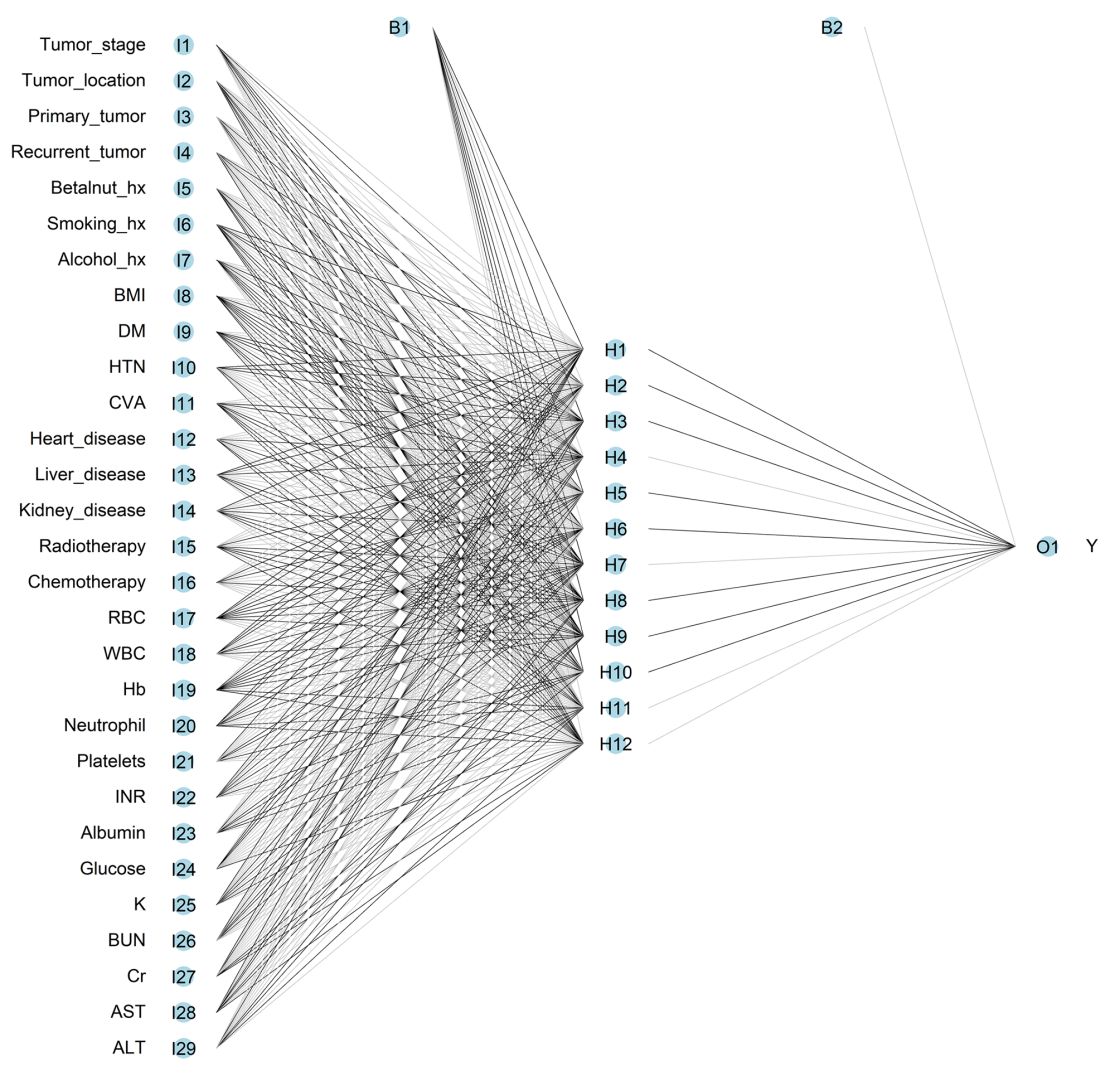
Architecture of feed-forward neural network for pre-operative prediction of surgical site infection in patients receiving free-flap reconstruction after head and neck cancer surgery The circles represent neurons, and the lines between circles represent modifiable connections. hx = history; BMI = body mass index; DM = diabetes mellitus, HTN = hypertension, CVA = cerebral vascular accident; RBC = red blood cell; WBC = white blood cell, Hb = hemoglobin, INR = international normalized ratio; K = potassium; BUN = blood urine nitrogen; Cr = creatinine; AST = aspartate aminotransferase; ALT = alanine aminotransferase.

**Figure 2:**
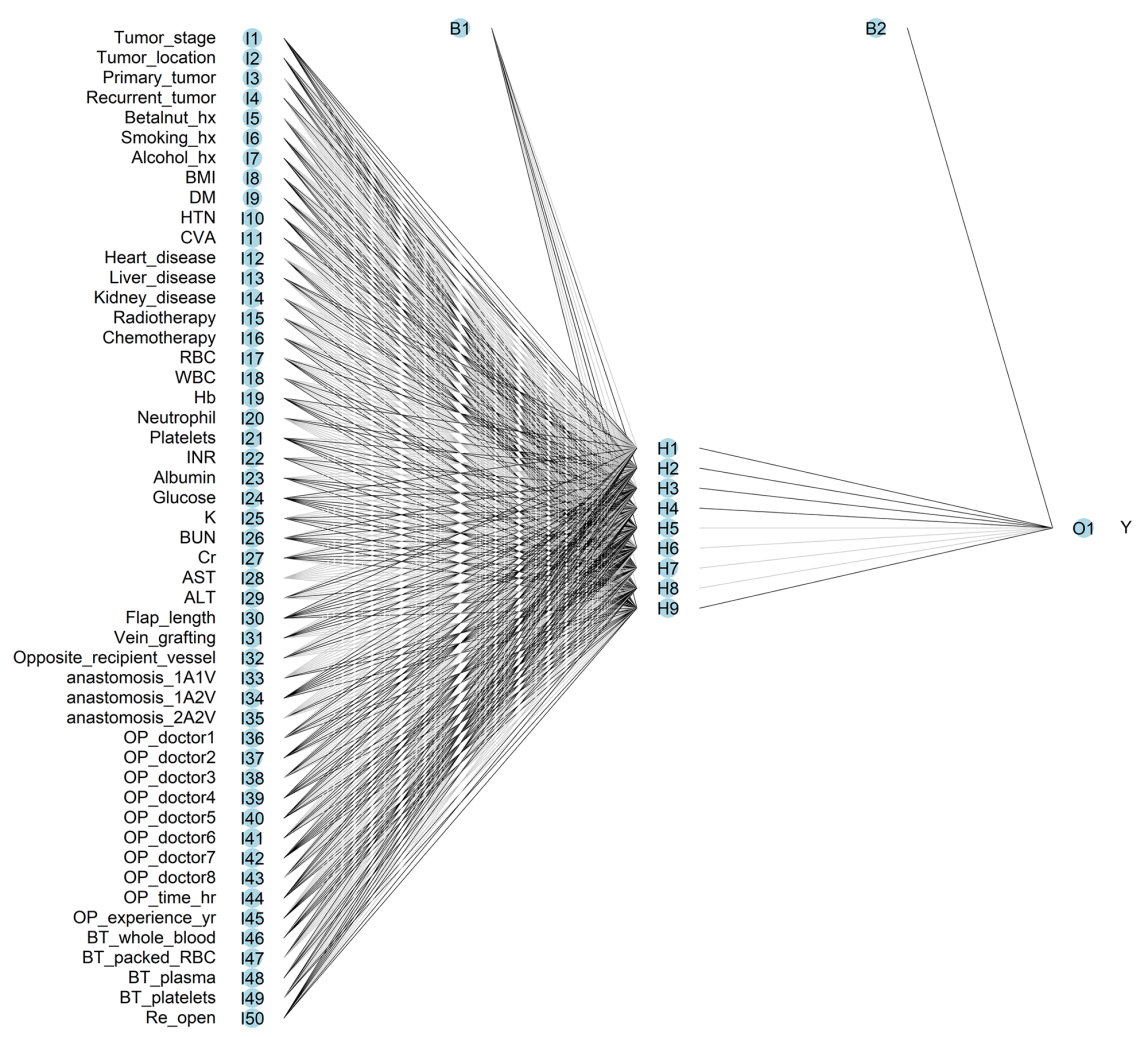
Architecture of feed-forward neural network for post-operative prediction of surgical site infection in patients receiving free-flap reconstruction after head and neck cancer surgery The circles represent neurons, and the lines between circles represent modifiable connections. hx = history; BMI = body mass index; DM = diabetes mellitus, HTN = hypertension, CVA = cerebral vascular accident; RBC = red blood cell; WBC = white blood cell, Hb = hemoglobin, INR = international normalized ratio; K = potassium; BUN = blood urine nitrogen; Cr = creatinine; AST = aspartate aminotransferase; ALT = alanine aminotransferase. 1A1V, 1A2V, and 2A2V indicated the anastomosed vessels were one artery one vein, one artery two veins, and two arteries two veins, respectively; OP= operator; BT = blood transfusion.

The constructed ANN model in pre-operative prediction includes 29 inputs, one bias neuron in the input layer, 12 hidden neurons, one bias neuron in the hidden layer, and one output neuron (Figure 1). The constructed ANN model in pre-operative prediction includes 50 inputs, one bias neuron in the input layer, 12 hidden neurons, one bias neuron in the hidden layer, and one output neuron (Figure 2). The accuracy, sensitivity, and specificity of the ANN in the pre-operative prediction of the training set were 81.00%, 60.90%, and 89.04%, respectively. The accuracy, sensitivity, and specificity of the ANN in post-operative prediction of the training set were 88.37%, 71.28%, and 95.21%, respectively. In the test set, the accuracy, sensitivity, and specificity of the ANN in the pre-operative and post-operative prediction were 77.8 ± 0.4%, 61.4 ± 0.8%, and 89.0 ± 0.4% as well as 75.7 ± 0.6%, 67.0 ± 1.5%, and 95.2 ± 0.2%, respectively (Table 4).

### Comparison between ANN and LR

**Figure 3:**
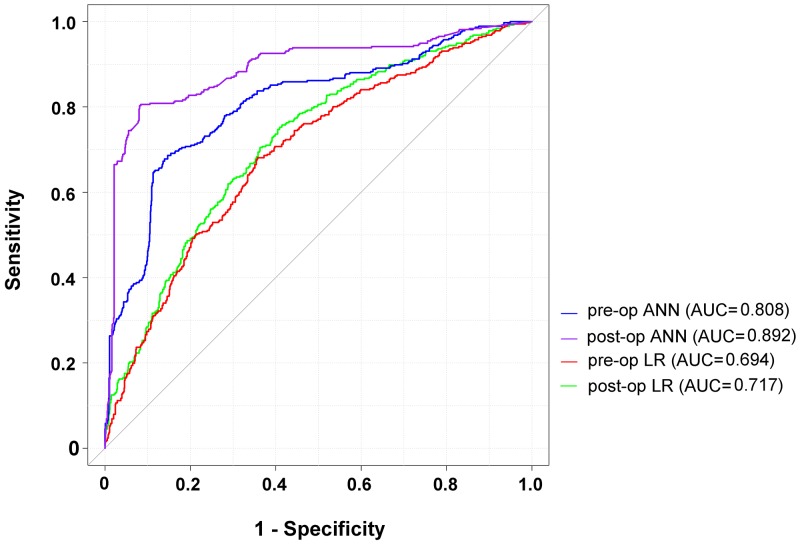
ROC curves for LR and ANN in pre-operative and post-operative prediction of the surgical site infection in patients receiving free-flap reconstruction after head and neck cancer surgery ROC = receiver operator characteristic; LR = logistic regression; ANN = artificial neural networks.

**Figure 4:**
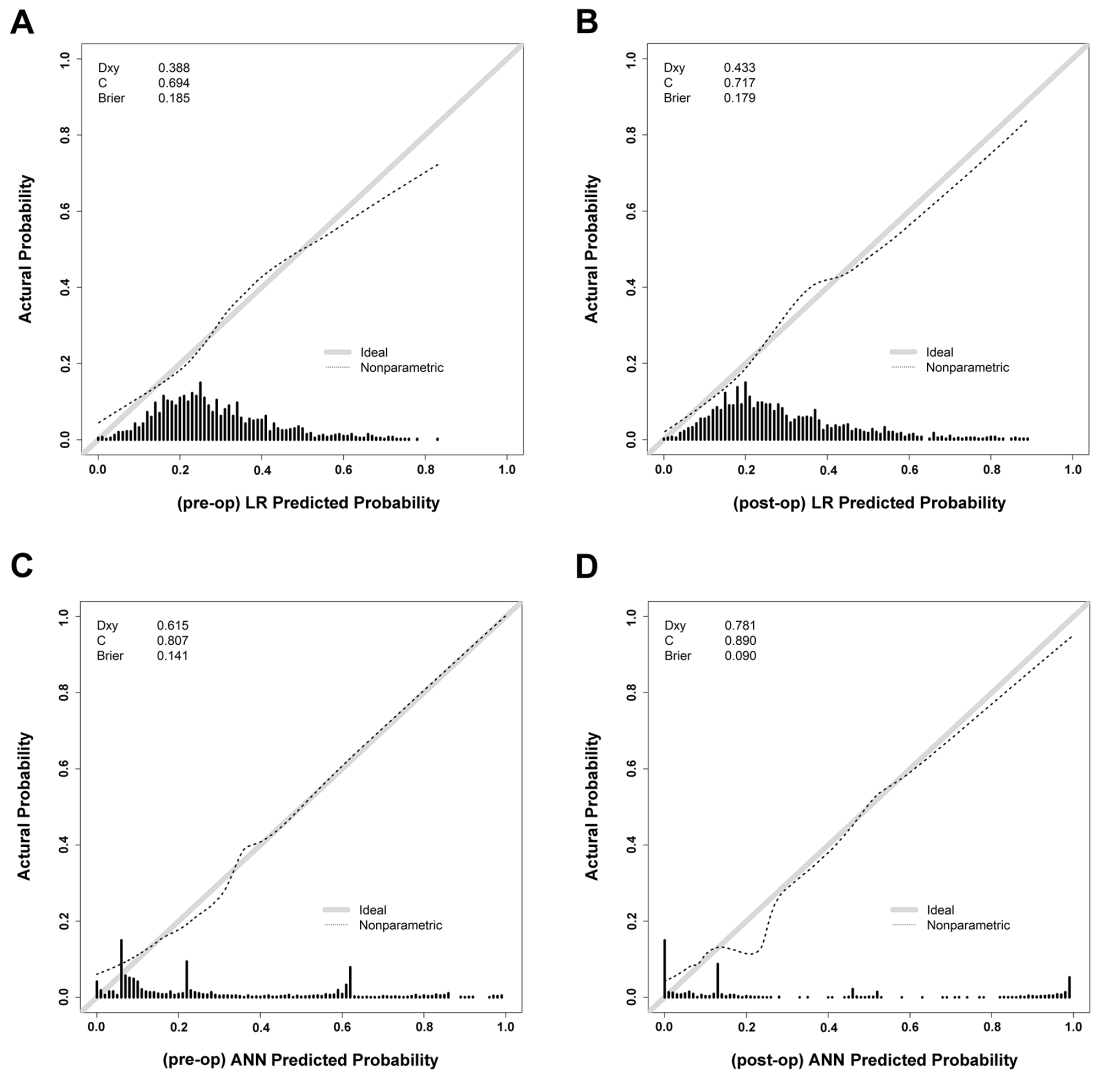
The calibration curves of the preoperative and postoperative predictions by LR and ANN

Because the higher rate of patients without SSIs than those with SSIs would be accompanied by a high accuracy and specificity in the prediction of SSI, therefore, we would rather focus on the sensitivity of these two models. In this study, ANN retained significantly higher sensitivity in pre-operative and post-operative prediction of the test set than LR (61.4% vs. 14.4% and 67.0% vs. 22.1%, respectively). In comparing AUCs of the ROCs between LR and ANN for the training set (Figure 3), the ANN had a significantly higher AUC (0.892) of post-operative prediction and AUC (0.808) of pre-operative prediction than AUC (0.7122) of post-operative prediction and AUC (0.694) of pre-operative prediction of LR (Table 5). The results suggest that the ANN has better performance than LR in either pre-operative or post-operative prediction. In addition, there was statistically significant higher AUC of post-operative prediction than pre-operative prediction by ANN (p<0.0001). The calibration curves of these four predictions by LR or ANN all plotted a nonparametric line close along the ideal diagonal line (Figure 4). With an agreement between these performance measures as the highest AUC, Dxy (0.781), c-index (0.890) and the lowest Brier score (0.090), the post-operative prediction by ANN had the highest overall predictive performance (Table 6).

**Table 5:** Statistical p-value among AUC comparisons between LR and ANN in the pre-operative and post-operative prediction

*P*-value	(Post-op) LR	(Pre-op) LR	(Pre-op) ANN	(Post-op) ANN
**(Post-op) LR**	-	0.0070	<0.0001	<0.0001
**(Pre-op) LR**	-	-	<0.0001	<0.0001
**(Pre-op) ANN**	-	-	-	<0.0001

AUC=area under the curve; LR=logistic regression; ANN=artificial neural networks.

**Table 6:** Assessment of predictive performance of LR and ANN in the pre-operative and post-operative prediction

	AUC	Dxy	C-index	Brier
(Pre-op) LR	0.694	0.388	0.694	0.185
(Post-op) LR	0.717	0.433	0.717	0.179
(Pre-op) ANN	0.808	0.615	0.807	0.141
(Post-op) ANN	0.892	0.781	0.890	0.090

LR=logistic regression; ANN=artificial neural networks.

## DISCUSSION

In this study, there were many interactions among the variables used for predicting SSI. For example, preoperative radiation causes fibrosis and scarring resulting in high rates of post-operative complications [34]. Scarring and fibrosis found in post-irradiation sites is associated with a longer time of operation [11]. In addition, osteoradionecrosis occurs in 1 to 6% of patients who receive radiation therapy to the head and neck region and is associated with a significant higher postoperative wound infection rate [8]. However, the general medical condition in patients selected for radiotherapy is generally considered to be reduced [35], but the number of comorbid illnesses increases as age increases [36]. Furthermore, generally more experienced surgeons perform surgery on patients who underwent previous radiotherapy [11]. Some authors advocate the contralateral neck vessels for microsurgical reconstruction [37], but the surgical time and elevated risk of kinking or compression of the vein is increased [38]. The need for microsurgical revision was also significantly higher [11], and the microsurgical revision is strongly associated with SSIs. LR can incorporate complex relationships with exceptional performance only if they are explicitly identified and by a relatively small set of independent predictors that fit the logistic model assumptions well [39]. However, the limitations of traditional LR become apparent when analyzing a complex dataset with many predictors, making it difficult to specify all possible interactions [40]. Only under conditions with relatively few variables (i.e., < 20), LR can provide odds ratio estimates for risk factors [41]. The characteristics of patients and diseases as well as the operations performed are various and present with unexploited interactions to predict SSI. In this study, the sensitivity of traditional LR for SSI prediction is extremely low (all less than or around 20%) in this study and cannot be used in the clinical setting. To improve the prediction performance, the non-linearity handled model (e.g., restricted cubic splines) [42, 43] or newly-developed network-regularized LR [44] may have a greater potential than the conventional LR for SSI prediction.

In contrast, establishing an ANN would require less domain knowledge than that required to develop an LR. As a dynamic approach to analyzing risk factors, ANN can modify internal structure to achieve a functional objective and give prediction outputs. With the computational power derived from the distributed nature of its connections, ANN can avoid dimensionality problems and successfully manages complex datasets, even when the ratio between variables is unbalanced or the sample size is small [45, 46]. Since no prior knowledge of the underlying data is required [39], ANN is ideally suited to deal with complex or unclear relationships of non-linear variables and recognizes patterns in sparse and noisy data making it a natural modeling tool to predict outcome in diverse populations [47]. Furthermore, ANN has been shown to be more accurate and to have better overall performance than LR in many clinical settings, such as to predict in-hospital mortality for patients with trauma injuries [48], patients receiving mechanical ventilation [49] as well as for patients in critical care [50].

However, unlike that LR is easier to generate confidence intervals in the model to perform area estimates of risk and have better clinical or real-life interpretation [39], one criticism of ANN is that it is difficult to assess the relative contribution of each variable to the final prediction put forth by the model [29, 51]. Additionally, ANN works as black box and does not provide detailed hazard ratio to indicate the direction and magnitude of influence of each variable on the outcome [52]. For example, in this study, age and Na level were not included as variables during the establishment of the ANN model, because their input would remarkably decrease the accuracy and sensitivity of prediction. However, the reason for such impairment is unknown. In addition, with simpler relationships between the predictor and outcome variables, LR is less prone to overfitting than ANN.

The performance in prediction of SSI by ANN is higher in post-operative than pre-operative predictions. Practically, the preoperative prediction by ANN can be used to facilitate a work to decrease SSI before the operation. However, there was still less satisfying prediction of SSI by ANN, considering the relatively low sensitivity of more than 60%. With additional information regarding the surgery, the postoperative prediction by ANN could provide a better predictive performance. In this study, the lack of potential SSI-related information, including oral hygiene and bacterial flora in the oral cavity [53], perioperative antibiotic use [54], existence of muscle portion of the flap [21], existence of prior osteoradionecrosis, requirement of mandibulectomy and the use of plate for bone fixation, which tend to create three-dimensional dead spaces and contribute to SSI [20], the status of nutrition, and postoperative wound management, may have rendered the prediction model a space for improvement. Furthermore, some of the factors measured were dichotomous variables rather than continuous variables, without considering dose response relationship between exposure levels of these risk factors and SSI. If available, the addition of such information may help increase ANN performance in mortality prediction.

Some other limitations of this study should be mentioned. First, the present study has the same limitations as any retrospective study: it is not controlled or randomized. Second, patients with a failed flap were excluded from analysis. However, because severe SSI may result in flap failure, such exclusion may result in a selection bias. Third, the imputation of laboratory data, particularly the glucose level, collected from the time prior to operation may not reflect changes in hemodynamics and the therapeutic effect of this variable. Furthermore, patients in the training and test sets were all from the same population. The predictive power of ANN was not validated in other populations, and therefore its generalizability could not be correctly determined. Finally, there is lack of uniformity of criteria for defining the SSI [55]. For example, the orocutaneous fistulas that did not meet the CDC criteria were not categorized as an SSI in some studies [56, 57]. However, some authors suggested that orocutaneous fistula caused by apparent infection such as abscess formation should be categorized as post-operative SSI [55]. The selection of different definition of SSI may cause a bias in the study.

Despite the limitations, it was a first step in showing the predictive power of SSI by ANN after free-flap reconstruction in patients receiving head and neck cancer surgery. ANN had a significantly higher predictive performance than the conventionally used LR and shed a light on a possible clinical application in the future.

## MATERIALS AND METHODS

### Ethical approval

This study was approved before its proceeding by the Institutional Review Board (IRB) of Chang Gung Memorial Hospital, a 2686-bed facility and Level I regional trauma center (approval number, 201700336B0). Informed consent was waived according to the regulations of the IRB.

### Subject and data preparation

Detailed information of 2,004 patients between March 2008 and February 2017 was retrieved from the registered free flap database and medical records of the hospital. In this study, the patient cohort included those who received free-flap reconstruction after head and neck cancer surgery. Patients with failure of the flap (n=107) or missing data (n=59) were not included in the dataset for analysis. Finally, 1,838 patients with 1,854 free-flap reconstructions were enrolled, with 16 patients receiving two-flap reconstruction simultaneously. The enrolled 1,854 reconstructions were randomly assigned in ratio of 7:3 into a training set (*n* = 1298) for predictor discovery and generation of a plausible model under supervised classification and a test set (*n* = 538) to test the performance of the model created in the training sample, respectively. The diagnosis of SSI was mainly according to the criteria of U.S. Centers for Disease Control and Prevention (CDC) [30], which classified SSIs into superficial incisional, deep incisional, and organ/space infection. In this study, purulent drainage, organisms identified from aseptically obtained specimens, spontaneous dehiscence with fever (>38°C) or localized pain or tenderness, and an abscess involving the wound bed found either on direct examination or by computed tomography examinations are indicators of SSI. In this study, the indications for SSIs that different from the CDC criteria included a clinical diagnosis of a wound infection by the operative surgeon was assigned as SSI; in addition, the orocutaneous fistula was not deemed as SSI, except there was evidence of prior wound infection. These patients were monitored closely postoperatively for signs of infection during daily examinations until the patient was discharged. The diagnosis of SSI was confirmed by the operative surgeon and then was recorded into our microsurgery registry system by one responsible nurse practitioner. The retrieved patient information included the following variables: age, sex, tumor stage, tumor location (data was arbitrarily divided into simple reconstruction after previous cancer ablation; regions of the lip, gum, buccal, and palate; regions of the mouth floor, tongue, and trigon; regions of the oropharyngeal and hypopharyngeal areas), conditions of resulted defect that requires a free-flap reconstruction (simple reconstruction after previous cancer ablation, defect after surgery for primary cancer, and defect after surgery for recurrent cancer), history of betel nut chewing, history of smoking, history of alcohol drinking, body mass index (BMI), pre-existed co-morbidities (such as DM, hypertension (HTN), cerebral vascular accident (CVA), heart diseases (ICD-9 code of 402, 410-416, and 420-429), liver diseases (ICD-9 code of 571), and renal diseases (ICD-9 code of 403-405 and 580-589)), pre-operative radiotherapy, and pre-operative chemotherapy. Blood-drawn laboratory data, including white blood cell count (WBC), red blood cell count (RBC), hemoglobin (Hb), hematocrit (Hct), percentage of neutrophil, platelets, international normalized ratio (INR), albumin, glucose, sodium (Na), potassium (K), blood urine nitrogen (BUN), creatinine (Cr), alanine aminotransferase (ALT), aspartate aminotransferase (AST), was also collected. Considering the perioperative data, a microsurgical failure that required a re-open operation may present a considerable effect on the post-operative SSI. In this study, the prediction of SSI was made at two time points, the pre-operative and post-operative stage. The prediction in the post-operative stage included information collected during perioperative stage. The perioperative data included flap length, usage of vein graft, anastomosed vessels (one artery one vein [1A1V], one artery two veins [1A2V], and two arteries two veins [2A2V]), surgeon who performed the reconstruction (indicated as operative doctor 1 to 8, those doctors who had the experience in less than 50 free-flap reconstruction would be assigned into the category of other doctors), operative time (hour), operative experience of the surgeon (years), amount of whole blood, packed RBC, plasma, or platelets in blood transfusion, type of the flaps, and re-open for a post-operative microsurgical failure.

### Multivariate logistic regression models

In this study, the LR classifier used glm function in the stats package in R3.3.3 (R Foundation for Statistical Computing, Vienna, Austria). A univariate LR analysis was initially performed to identify significant predictors of SSI. To develop a good-fit model, all significant variables derived from univariate analysis were entered into the model. Variables with 5% significance were included in the multiple LR using stepwise elimination to identify independent risk factors for SSI. A prediction model was developed using the probability value calculated from summary score assigned to final variables based on its regression coefficient.

### Artificial neural network (ANN) model

In this study, the ANN classifier used the ‘‘nnet’’ algorithm, which is a feed-forward neural network, and multinomial log-linear models, with the nnet function in the nnet package in R. The models contained three layers: an input layer, a single output node, and a single layer of hidden nodes. The number of hidden layer neurons was determined through trial and error, since no accepted theory currently exists for predetermining the optimal number of hidden layer neurons. The number of hidden layer neurons was selected to lead to a predictive network with the best sensitivity and specificity. Tuning parameters included the number of nodes in the hidden layer optimized between 1 and 20. For the training process, maximal iterations and decay were selected as 1000 and 0.001, respectively. To avoid over-fitting, iterations occurred until the error did not significantly decrease.

### Performance of ANN and LR

The accuracy, sensitivity, and specificity of the ANN and LR models were calculated. Stratified 10-fold cross-validation was used to evaluate the predictive power of the models. Briefly, the patients were randomly divided into 10-folds with the number of patients with an event approximately equal in all folds. The model was developed using 9-folds and validation on the tenth. Measures of model performance regarding the area under the curve (AUC) of the receiver operator characteristic curves (ROCs), Somers’ Dxy rank correlation coefficient, c-index, calibration curve, and Brier score corresponding to the two different models were measured. A nonparametric approach was performed to analyze the AUC under correlated ROCs using the roc & roc.test function in the pROC package in R, as this approach allowed for the correlated nature of the data to be taken into account such that two or more empirical curves could be constructed based on tests performed on the same individuals [31]. The predicted probabilities against binary events was validated using the val.prob function in the rms package in R. Somers’ Dxy assess the predictive discrimination with measured probability of concordance minus the probability of discordance between predicted outcomes and observed outcomes [32]. C-index show how well the model can discriminate between those who have SSI and those who have not; a c-index of 0.5 indicates that the model is useless in predicting SSI and a value of 1.0 suggests perfect discrimination. A calibration curve plots to indicate the agreement between the predicted probabilities and observed outcomes. The Brier score is defined as the mean squared error between the predicted probabilities and the actual outcomes and can be considered as an overall measure of model performance [33]. Brier scores vary between 0 and 1, a lower score indicating higher accuracy.

### Statistical analyses

All statistical analyses were performed using SPSS 20.0 (IBM Inc., Chicago, IL, USA) and R 3.3.3. We used Chi-square tests to determine the significance of the association between categorical variables. For continuous variables, we used Mann-Whitney *U* tests to compare distributed data. Results are presented as mean ± standard deviation, and a p-value of < 0.05 was considered statistically significant.

## CONCLUSION

We demonstrated that The post-operative prediction by ANN had the highest overall performance in predicting SSI after free-flap reconstruction in patients receiving surgery for head and neck cancer. The results of studies published so far are encouraging and may provide the first steps towards the development of a prediction model to be used in patient care and reduce occurrence of such postoperative complication.

## References

[1] Hsieh CH, Yang JC, Chen CC, Kuo YR, Jeng SF (2009). Alternative reconstructive choices for anterolateral thigh flap dissection in cases in which no sizable skin perforator is available. Head Neck.

[2] Spyropoulou GA, Jeng SF, Hsieh CH, Tsimponis A, Shih HS (2014). Microsurgical reconstruction for head and neck cancer in elderly patients. J Reconstr Microsurg.

[3] Karakida K, Aoki T, Ota Y, Yamazaki H, Otsuru M, Takahashi M, Sakamoto H, Miyasaka M (2010). Analysis of risk factors for surgical-site infections in 276 oral cancer surgeries with microvascular free-flap reconstructions at a single university hospital. J Infect Chemother.

[4] Penel N, Fournier C, Lefebvre D, Lefebvre JL (2005). Multivariate analysis of risk factors for wound infection in head and neck squamous cell carcinoma surgery with opening of mucosa. Study of 260 surgical procedures. Oral Oncol.

[5] Penel N, Lefebvre D, Fournier C, Sarini J, Kara A, Lefebvre JL (2001). Risk factors for wound infection in head and neck cancer surgery: a prospective study. Head Neck.

[6] Sepehr A, Santos BJ, Chou C, Karimi K, Devcic Z, Oels S, Armstrong WB (2009). Antibiotics in head and neck surgery in the setting of malnutrition, tracheotomy, and diabetes. Laryngoscope.

[7] Liljemark WF, Bloomquist C (1996). Human oral microbial ecology and dental caries and periodontal diseases. Crit Rev Oral Biol Med.

[8] Agra IM, Carvalho AL, Pontes E, Campos OD, Ulbrich FS, Magrin J, Kowalski LP (2003). Postoperative complications after en bloc salvage surgery for head and neck cancer. Arch Otolaryngol Head Neck Surg.

[9] de Melo GM, Ribeiro KC, Kowalski LP, Deheinzelin D (2001). Risk factors for postoperative complications in oral cancer and their prognostic implications. Arch Otolaryngol Head Neck Surg.

[10] Lee DH, Kim SY, Nam SY, Choi SH, Choi JW, Roh JL (2011). Risk factors of surgical site infection in patients undergoing major oncological surgery for head and neck cancer. Oral Oncol.

[11] Mucke T, Rau A, Weitz J, Ljubic A, Rohleder N, Wolff KD, Mitchell DA, Kesting MR (2012). Influence of irradiation and oncologic surgery on head and neck microsurgical reconstructions. Oral Oncol.

[12] Dormand EL, Banwell PE, Goodacre TE (2005). Radiotherapy and wound healing. Int Wound J.

[13] Ferrier MB, Spuesens EB, Le Cessie S, Baatenburg de Jong RJ (2005). Comorbidity as a major risk factor for mortality and complications in head and neck surgery. Arch Otolaryngol Head Neck Surg.

[14] Schwartz SR, Yueh B, Maynard C, Daley J, Henderson W, Khuri SF (2004). Predictors of wound complications after laryngectomy: a study of over 2000 patients. Otolaryngol Head Neck Surg.

[15] Szlosek DA, Ferrett J (2016). Using machine learning and natural language processing algorithms to automate the evaluation of clinical decision Support in electronic medical record systems. EGEMS (Washington, DC).

[16] Malone DL, Genuit T, Tracy JK, Gannon C, Napolitano LM (2002). Surgical site infections: reanalysis of risk factors. J Surg Res.

[17] Ma CY, Ji T, Ow A, Zhang CP, Sun J, Zhou XH, Wang LZ, Sun KD, Han W (2012). Surgical site infection in elderly oral cancer patients: is the evaluation of comorbid conditions helpful in the identification of high-risk ones?. J Oral Maxillofac Surg.

[18] Kafaki SB, Alaedini K, Qorbani A, Asadian L, Haddadi K (2016). Hyperglycemia: a predictor of death in severe head injury patients. Clin Med Insights Endocrinol Diabetes.

[19] Paydarfar JA, Birkmeyer NJ (2006). Complications in head and neck surgery: a meta-analysis of postlaryngectomy pharyngocutaneous fistula. Arch Otolaryngol Head Neck Surg.

[20] Kamizono K, Sakuraba M, Nagamatsu S, Miyamoto S, Hayashi R (2014). Statistical analysis of surgical site infection after head and neck reconstructive surgery. Ann Surg Oncol.

[21] Liu SA, Wong YK, Poon CK, Wang CC, Wang CP, Tung KC (2007). Risk factors for wound infection after surgery in primary oral cavity cancer patients. Laryngoscope.

[22] Yahya N, Ebert MA, Bulsara M, House MJ, Kennedy A, Joseph DJ, Denham JW (2016). Statistical-learning strategies generate only modestly performing predictive models for urinary symptoms following external beam radiotherapy of the prostate: a comparison of conventional and machine-learning methods. Med Phys.

[23] Langness S, Costantini TW, Smith A, Bansal V, Coimbra R (2017). Isolated traumatic brain injury in patients with cirrhosis: do different treatment paradigms result in increased mortality?. Am J Surg.

[24] Belusic-Gobic M, Car M, Juretic M, Cerovic R, Gobic D, Golubovic V (2007). Risk factors for wound infection after oral cancer surgery. Oral Oncol.

[25] Chen ZY, Liu JH, Liang K, Liang WX, Ma SH, Zeng GJ, Xiao SY, He JG (2012). The diagnostic value of a multivariate logistic regression analysis model with transvaginal power doppler ultrasonography for the prediction of ectopic pregnancy. J Int Med Res.

[26] Adavi M, Salehi M, Roudbari M (2016). Artificial neural networks versus bivariate logistic regression in prediction diagnosis of patients with hypertension and diabetes. Med J Islam Repub Iran.

[27] Manning T, Sleator RD, Walsh P (2014). Biologically inspired intelligent decision making: a commentary on the use of artificial neural networks in bioinformatics. Bioengineered.

[28] Zhang Z (2016). A gentle introduction to artificial neural networks. Ann Transl Med.

[29] Tu JV (1996). Advantages and disadvantages of using artificial neural networks versus logistic regression for predicting medical outcomes. J Clin Epidemiol.

[30] Mangram AJ, Horan TC, Pearson ML, Silver LC, Jarvis WR, Centers for Disease Control and Prevention (CDC) Hospital Infection Control Practices Advisory Committee (1999). Guideline for prevention of surgical site infection, 1999. Centers for disease control and prevention (CDC) hospital infection control practices a dvisory committee. Am J Infect Control.

[31] DeLong ER, DeLong DM, Clarke-Pearson DL (1988). Comparing the areas under two or more correlated receiver operating characteristic curves: a nonparametric approach. Biometrics.

[32] Harrell FE, Lee KL, Mark DB (1996). Multivariable prognostic models: issues in developing models, evaluating assumptions and adequacy, and measuring and reducing errors. Stat Med.

[33] Brier GW (1950). Verification of forecasts expressed in terms of probability. Mon Weather Rev.

[34] Stoeckli SJ, Pawlik AB, Lipp M, Huber A, Schmid S (2000). Salvage surgery after failure of nonsurgical therapy for carcinoma of the larynx and hypopharynx. Arch Otolaryngol Head Neck Surg.

[35] Epstein JB, Emerton S, Kolbinson DA, Le ND, Phillips N, Stevenson-Moore P, Osoba D (1999). Quality of life and oral function following radiotherapy for head and neck cancer. Head Neck.

[36] Uçkay I, Harbarth S, Peter R, Lew D, Hoffmeyer P, Pittet D (2010). Preventing surgical site infections. Expert Rev Anti Infect Ther.

[37] Head C, Sercarz JA, Abemayor E, Calcaterra TC, Rawnsley JD, Blackwell KE (2002). Microvascular reconstruction after previous neck dissection. Arch Otolaryngol Head Neck Surg.

[38] Hanasono MM, Barnea Y, Skoracki RJ (2009). Microvascular surgery in the previously operated and irradiated neck. Microsurgery.

[39] Tang ZH, Liu J, Zeng F, Li Z, Yu X, Zhou L (2013). Comparison of prediction model for cardiovascular autonomic dysfunction using artificial neural network and logistic regression analysis. PLoS One.

[40] Gu W, Vieira AR, Hoekstra RM, Griffin PM, Cole D (2015). Use of random forest to estimate population attributable fractions from a case-control study of salmonella enterica serotype enteritidis infections. Epidemiol Infect.

[41] Knol MJ, Vandenbroucke JP, Scott P, Egger M (2008). What do case-control studies estimate? Survey of methods and assumptions in published case-control research. Am J Epidemiol.

[42] Gu W, Ren Y, Ji L, Hong T, Mu Y, Guo L, Li Q, Tian Q, Yang X (2016). Non-linear associations of risk factors with mild hypoglycemia among chinese patients with type 2 diabetes. J Diabetes Complications.

[43] Churpek MM, Yuen TC, Winslow C, Meltzer DO, Kattan MW, Edelson DP (2016). Multicenter comparison of machine learning methods and conventional regression for predicting clinical deterioration on the wards. Crit Care Med.

[44] Zhang W, Wan YW, Allen GI, Pang K, Anderson ML, Liu Z (2013). Molecular pathway identification using biological network-regularized logistic models. BMC Genomics.

[45] Zou J, Han Y, So SS (2008). Overview of artificial neural networks. Methods Mol Biol.

[46] Banner MJ, Euliano NR, Brennan V, Peters C, Layon AJ, Gabrielli A (2006). Power of breathing determined noninvasively with use of an artificial neural network in patients with respiratory failure. Crit Care Med.

[47] Becalick DC, Coats TJ (2001). Comparison of artificial intelligence techniques with UKTRISS for estimating probability of survival after trauma. UK trauma and injury severity score. J Trauma.

[48] DiRusso SM, Sullivan T, Holly C, Cuff SN, Savino J (2000). An artificial neural network as a model for prediction of survival in trauma patients: validation for a regional trauma area. J Trauma.

[49] Chen CJ, Shi HY, Lee KT, Huang TY (2013). In-hospital mortality prediction in patients receiving mechanical ventilation in taiwan. Am J Crit Care.

[50] Kim S, Kim W, Park RW (2011). A comparison of intensive care unit mortality prediction models through the use of data mining techniques. Healthc Inform Res.

[51] Patel JL, Goyal RK (2007). Applications of artificial neural networks in medical science. Curr Clin Pharmacol.

[52] Chiu HC, Ho TW, Lee KT, Chen HY, Ho WH (2013). Mortality predicted accuracy for hepatocellular carcinoma patients with hepatic resection using artificial neural network. Sci World J.

[53] Hirakawa H, Hasegawa Y, Hanai N, Ozawa T, Hyodo I, Suzuki M (2013). Surgical site infection in clean-contaminated head and neck cancer surgery: risk factors and prognosis. Eur Arch Otorhinolaryngol.

[54] Khariwala SS, Le B, Pierce BH, Vogel RI, Chipman JG (2016). Antibiotic use after free tissue reconstruction of head and neck defects: short course vs. Long Course. Surg Infect (Larchmt).

[55] Akashi M, Kusumoto J, Sakakibara A, Hashikawa K, Furudoi S, Komori T (2017). Literature review of criteria for defining recipient-site infection after oral oncologic surgery with simultaneous reconstruction. Surg Infect (Larchmt).

[56] Durand ML, Yarlagadda BB, Rich DL, Lin DT, Emerick KS, Rocco JW, Deschler DG (2015). The time course and microbiology of surgical site infections after head and neck free flap surgery. Laryngoscope.

[57] Yarlagadda BB, Deschler DG, Rich DL, Lin DT, Emerick KS, Rocco JW, Durand ML (2016). Head and neck free flap surgical site infections in the era of the surgical care improvement project. Head Neck.

